# Geographic and ancestral variation in the association between *APOE* ε4 and cognitive function in South Asians from LASI‐DAD

**DOI:** 10.1002/alz.092646

**Published:** 2025-01-03

**Authors:** Yi Zhe Wang, Wei Zhao, Elise Kerdoncuff, Zheng Li, Priya Moorjani, Alden L. Gross, Xiang Zhou, Sharmistha Dey, Aparajit Ballav Dey, Jinkook Lee, Jennifer A Smith, Sharon L R Kardia

**Affiliations:** ^1^ University of Michigan School of Public Health, Ann Arbor, MI USA; ^2^ University of Michigan Institute for Social Research, Ann Arbor, MI USA; ^3^ University of California, Berkeley, Berkeley, CA USA; ^4^ Johns Hopkins Bloomberg School of Public Health, Baltimore, MD USA; ^5^ All India Institute of Medical Sciences, New Delhi India; ^6^ All India Institute of Medical Sciences, New Delhi, New Delhi, Delhi India; ^7^ University of Southern California, Los Angeles, CA USA

## Abstract

**Background:**

The ε4 allele of the apolipoprotein E (*APOE*) gene is the strongest genetic determinant for Alzheimer’s disease and cognitive function in nearly all human populations, yet inconsistent effects have been reported in South Asians. The population of India has admixed genetic ancestry with most people falling on a North/South cline and having varying proportions of Ancestral North Indian (ANI) and Ancestral South Indian (ASI) ancestries, and those in east of India fall off the cline due to ancestry from additional ancestral populations. This study examined the ε4 association with cognitive function across 18 states/union territories of India and investigated whether ancestral background modulates ε4 association with cognitive function in 2,590 participants from the Longitudinal Aging Study in India – Diagnostic Assessment of Dementia (LASI‐DAD).

**Method:**

*APOE* ε4 carrier status was determined from rs429358 and rs7412 genotypes measured by TaqMan Assays. Percent ANI ancestry (%ANI) was estimated from whole genome sequencing for those in the North/South cline (range: 22‐80%, n = 2,097). We first investigated ε4 associations with seven cognitive measures, including five broad cognitive domain scores, general cognitive function, and the Hindi Mental State Examination (HMSE) score, within each state/union territory using linear regression. Subsequently, participants were categorized into ancestral groups based on %ANI tertile (low, medium, and high), along with an out‐of‐cline group. Effect modification by ancestry groups was assessed using cross‐product interaction terms and subgroup analysis. All models were adjusted for age, sex, state of residence (if applicable), education, literacy, urban/rural residence, caste, per capita household consumption, and the first 10 genetic principal components.

**Result:**

The ε4 effect varied significantly across states/union territories for general cognitive function, executive function, and language/fluency (Cochran’s Q test p<0.05, I^2^: 42.0‐57.7%). While ε4 by ancestral group interaction terms were not significant for any cognitive measure, stratified analysis showed a trend of a larger ε4 effects in the middle and high %ANI tertiles, with significant associations observed for HMSE score and memory within the highest %ANI tertile (p<0.05).

**Conclusion:**

Our results suggest that ancestral background may play a role in modulating the ε4 effects on cognitive function, contributing to the observed heterogeneity across Indian groups.

